# Systemic Administration of G-CSF Accelerates Bone Regeneration and Modulates Mobilization of Progenitor Cells in a Rat Model of Distraction Osteogenesis

**DOI:** 10.3390/ijms22073505

**Published:** 2021-03-28

**Authors:** Flavy Roseren, Martine Pithioux, Stéphane Robert, Laure Balasse, Benjamin Guillet, Edouard Lamy, Sandrine Roffino

**Affiliations:** 1 ISM, Aix Marseille University, CNRS, 13009 Marseille, France; flavy.ROSEREN@univ-amu.fr (F.R.); edouard.lamy@univ-amu.fr (E.L.); sandrine.roffino@univ-amu.fr (S.R.); 2 Department of Orthopaedics and Traumatology, Institute for Locomotion, Sainte-Marguerite Hospital, ISM, Aix Marseille University, APHM, CNRS, 13009 Marseille, France; 3 Anatomic Laboratory, ISM, Aix Marseille University, CNRS, 13005 Marseille, France; 4 Mecabio Platform, ISM, Aix Marseille University, 13009 Marseille, France; 5 AMUTICYT, C2VN, INRAE, INSERM, Aix Marseille University, 13005 Marseille, France; Stephane.robert@univ-amu.fr; 6 C2VN, CERIMED, Aix Marseille University, 13005 Marseille, France; laure.BALASSE@univ-amu.fr (L.B.); benjamin.guillet@univ-amu.fr (B.G.)

**Keywords:** neovascularization, endothelial progenitor cells, mesenchymal stromal cells, hematopoietic stem/progenitor cells, bone formation, G-CSF

## Abstract

Granulocyte colony-stimulating factor (G-CSF) was shown to promote bone regeneration and mobilization of vascular and osteogenic progenitor cells. In this study, we investigated the effects of a systemic low dose of G-CSF on both bone consolidation and mobilization of hematopoietic stem/progenitor cells (HSPCs), endothelial progenitor cells (EPCs) and mesenchymal stromal cells (MSCs) in a rat model of distraction osteogenesis (DO). Neovascularization and mineralization were longitudinally monitored using positron emission tomography and planar scintigraphy. Histological analysis was performed and the number of circulating HSPCs, EPCs and MSCs was studied by flow cytometry. Contrary to control group, in the early phase of consolidation, a bony bridge with lower osteoclast activity and a trend of an increase in osteoblast activity were observed in the distracted callus in the G-CSF group, whereas, at the late phase of consolidation, a significantly lower neovascularization was observed. While no difference was observed in the number of circulating EPCs between control and G-CSF groups, the number of MSCs was significantly lower at the end of the latency phase and that of HSPCs was significantly higher 4 days after the bone lengthening. Our results indicate that G-CSF accelerates bone regeneration and modulates mobilization of progenitor cells during DO.

## 1. Introduction

Distraction osteogenesis is a widely used surgical technique for the treatment of bone non-union, limb lengthening discrepancies, congenital deformities and filling long bone defects. Its principle is based on a gradual displacement of the bone segment part after an osteotomy [1]. DO is organized in three temporal phases: latency, active distraction, and consolidation. The latency period starts right after the osteotomy and ends before activation of the external device. During this phase, an inflammatory reaction occurs and contributes to the development of the callus. The second phase consists of stretching the new regenerate formed between the two cortices with a specific rate and rhythm until the desired length is reached. Finally, the consolidation phase ends when ceasing the distraction forces and mineralization of the distracted callus occurs, leading to an osseous union of the distracted gap [2]. In spite of its efficiency, DO can lead to numerus complications such as pseudarthrosis, infection, non-union and fractures [3,4,5,6]. Therefore, accelerating the bone regeneration is currently a challenge that would reduce the occurrence of these complications.

The DO regenerative process is driven by two natural events called osteogenesis and vascularization [2,7,8,9,10,11,12,13]. Osteogenesis and vascularization are led by the recruitment and migration to the distracted callus of progenitor cells from multiple sites, including the bone itself, along with the periosteal membrane, endosteum and bone marrow [14,15,16,17]. Then, these cells differentiate into a specific cell type depending on the environmental signals [2,18,19]. Mesenchymal stromal cells (MSCs) and hematopoietic stem/progenitor cells (HSPCs) act synergistically in osteogenesis. On the one hand, differentiation of HSPCs into monocytes [20] provides a reservoir of osteoclasts that remove the mineralized tissue (cartilage and bone) [21]. On the other hand, MSCs participate in bone tissue formation during DO [22] as they can differentiate into fibroblasts, chondroblasts or osteoblasts [7]. Moreover, endothelial progenitor cells (EPCs) are the main actor of neovascularization in skeletal repair [23]. These cells tend to migrate to regions of neovascularization to differentiate into mature endothelial cells and induce vasculogenesis [24]. During DO, it has been demonstrated that EPC mobilization is promoted during the latency phase, the distractive phase and at the early consolidation phase by the ischemic environment in the distracted callus and that EPCs homed to the distracted callus [25,26]. Finally, it is important to point out that interactions between osteogenesis and vascularization are crucial to allow bone formation since it has been shown that a lack of vascularization led to a delay in bone formation [12,18,27,28,29]. In addition, in a dog distraction model, hypoxia induced by the alteration of the micro-circulation encouraged MSC differentiation into chondroblasts instead of osteoblasts [12,29].

Cytokines like granulocyte colony-stimulating factor (G-CSF) permit the mobilization of stem and progenitor cells from the bone marrow niches to the peripheral blood stream [30]. By disturbing the CXCR4/SDF-1 pathway, G-CSF affects the interaction between adhesion molecules and their ligands, promoting the egress of HSPCs [31], MSCs [32,33] and EPCs [34] into the blood. In humans, G-CSF is used to induce HSPC mobilization in severe chronic neutropenic disease, to reduce chemotherapy-induced neutropenia as well as in healthy donors for allogenic hematopoietic cell transplantation for neutropenic patients [35,36].

While G-CSF has the faculty to mobilize HSPCs, EPCs and MSCs, its effect on bone regeneration has been investigated in only a few studies carried out in fracture, defect and non-union models [37,38,39,40]. G-CSF was shown to promote bone regeneration [37,39,40,41], but also to improve bone mechanical properties since stiffness and maximum failure force were increased compared to the control group in both high- [38] and low-dose administration models [39]. However, to date, there are no data on the impact of G-CSF on bone regeneration in DO. Furthermore, the mechanisms responsible for G-CSF’s positive effects on bone regeneration have not yet been elucidated. Only one study, in a model of critical size femoral defects in rats, suggested that G-CSF may have positive effects on bone repair by improving vascularization through the mobilization of CD34+ progenitor cells [40]. We hypothesize that G-CSF improves bone regeneration during DO through an increase in the mobilization of progenitor cells. Therefore, the goal of this study is to determine the effects of the systemic administration of a 5 µg/kg per day dose of G-CSF on both bone regeneration and mobilization kinetic patterns of HSPCs, EPCs and MSCs in a rat model of DO.

## 2. Results

### 2.1. G-CSF Altered Kinetic Patterns of Mobilization of HSPCs

**Figure 1:**
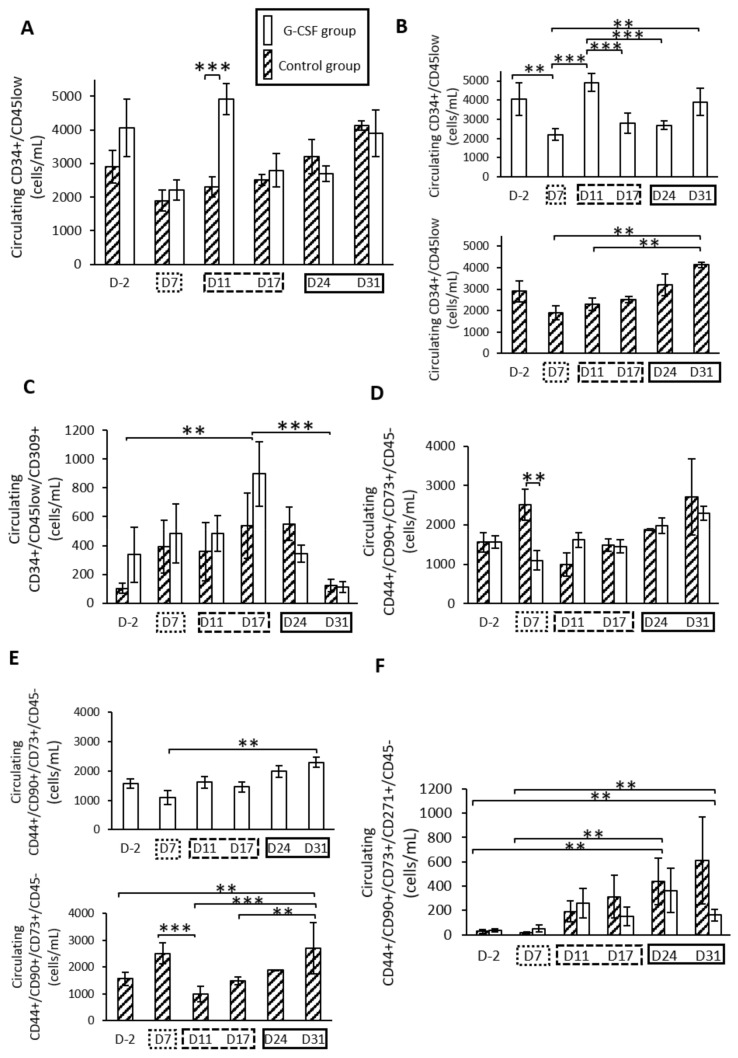
Mobilization of hematopoietic stem/progenitor cells (HSPCs) (**A**,**B**), endothelial progenitor cells (EPCs) (**C**) and mesenchymal stromal cells (MSCs) (**D**–**F**) during the three phases of distraction osteogenesis. (**A**) A significant peak of HSPC concentration at D11 for the granulocyte colony-stimulating factor (G-CSF) group compared to the control group. (**B**) Representation of intragroup mobilization patterns of HSPCs. (**C**) For EPC count, only an effect of time is observed, with a significant peak at D17. (**D**) A statistical difference in the mobilization pattern of total MSCs at an early time point (D7). (**E**) Intragroup kinetic pattern for MSC population. A marked augmentation of MSCs in the blood stream at 31 days post-surgery is effective for both groups. (**F**) For MSC-271+ cells, a statistical time effect is seen for later times (D24 and D31) compared to early time points. 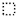 represents the latency period, the distraction phase is represented by 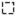, and 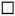 represents the consolidation phase. ** *p* < 0.05, *** *p* < 0.01. Cytometry assays were performed on 6 G-CSF animals and 4 control animals.

In the control group, a progressive increase in the HSPC level was observed throughout the protocol of DO (Figure 1). At the end of the latency period (D7), the HSPC level was at 1896 ± 315 cells/ml and continued to rise significantly (*p* < 0.05) to 4128 ± 135 cells/mL at 31 days post-surgery (D31) (Figure 1B). In the G-CSF group, HSPC level was significantly increased compared to control group at four days after lengthening (D11, *p* < 0.01) (Figure 1A). In this group, intragroup comparisons showed a significant difference between D11 and both D7 and D17 (*p* < 0.01). From D17 to D31, HSPC levels were similar between G-CSF and control groups (Figure 1B).

### 2.2. G-CSF Had No Effect on the Kinetic Pattern of Mobilization of EPCs

The kinetic pattern of EPCs was not different between the G-CSF and control groups. However, a significant time effect was observed throughout the distraction process (*p* < 0.004) (Figure 1C). At the end of the latency period (D7), a slight increase for each group was observed compared to the baseline level. The mobilization increased and peaked significantly at the end of the distraction phase (D17) for both groups, with 537 ± 225 cells/mL for the control group and 896 ± 222 cells/mL for the G-CSF group compared to the baseline level (D-2) (*p* < 0.05). The augmentation was more important for the G-CSF group, at 1.7-fold higher compared to the control group, even though no statistical difference was observed. Subsequently, EPC concentration decreased during the early consolidation period and reached a statistical difference at D17 (*p* < 0.01). The EPC count obtained a minimum value at 31 days post-surgery (D31) with 124 ± 43 cells/mL for the control group and 110 ± 39 cells/mL for the G-CSF group.

### 2.3. G-CSF Altered Kinetic Patterns of Mobilization of MSCs

The kinetic pattern of MSCs (Figure 1D) was different between the control and G-CSF groups (*p* = 0.022). In the control group, the MSC level increased at the end of the latency period (D7) compared to before surgery (D-2) (Figure 1E). After 4 days of distraction (D11), the MSC level (2512 ± 386 cells/mL) decreased significantly compared to the end of the latency period (986 ± 293 cells/mL). At 31 days post-surgery (D31), the MSC level was significantly increased compared to the levels obtained during the active distraction phase. In the G-CSF group (Figure 1E), the number of MSCs (1093 ± 247 cells/mL) was significantly lower at the end of the latency period (D7) compared to the control group (2512 ± 386 cells/mL) (*p* = 0.017) (Figure 1D). From D11 to D31, the kinetic pattern of MSCs was similar to the control group (Figure 1D).

When considering the population of less differentiated MSCs labeled with CD271, a similar mobilization pattern was observed throughout the protocol of DO in control and G-CSF groups (Figure 1F). A repeated ANOVA using a mixed model only showed a significant effect of the time (*p* = 0.002). A gradual increase was observed throughout the protocol of DO, with a maximum value at 31 days post-surgery (D31) (Figure 1F). The number of MSCs labeled with CD271 was significantly different between the early times (D2 and D7) compared to the later ones (D24 and D31) (*p* < 0.05).

### 2.4. Trend of Increase in Hydroxyapatite Deposition at 3 Weeks of Consolidation

**Figure 2:**
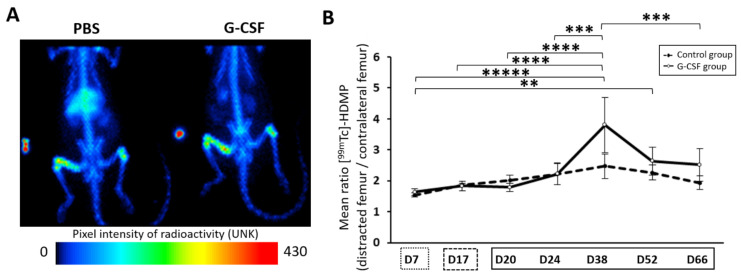
Assessment of bone metabolism throughout the protocol of distraction. (**A**) Representation of planar scintigraphy imaging at 38 days post-surgery. Maximum radioactivity values are indicated in red to orange, medium values in yellow to green and lowest values in blue to black (UNK unity). (**B**) The temporal pattern of the mean ratio uptake is presented and demonstrates osteoblast activity. The solid line corresponds to the injected group while the dashed line is for the control group. At D38, a time effect is apparent and differs from all time points except for D52. 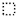 represents the latency period, 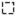 represents the distraction phase and the consolidation phase is represented by 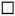. Planar scintigraphy was performed on 6 control animals and 4 G-CSF animals. ** *p* < 0.05, *** *p* < 0.01, **** *p* < 0.001, ***** *p* < 0.0001.

The continuous deposition of hydroxyapatite from the beginning of the regenerative process to the end of the study was monitored with Mediso-NanoSPET-CT. The accumulation by chemisorption of the radiotracer [^99m^Tc]-HDMP onto newly formed hydroxyapatite crystals allowed us to indirectly assess the osteoblast activity inside the distracted callus. A repeated ANOVA using a mixed model showed a significant effect of time (*p* < 0.0001). The mean ratio uptake of [^99m^Tc]-HDMP increased from the end of the latency period to D38. On this day, the mean ratio uptake reached a maximum and then slowly diminished. At the end of the assay, the mineral deposition was still effective (2.52 ± 0.5 and 1.94 ± 0.2 for G-CSF and control groups, respectively). There was no significant difference in hydroxyapatite deposition profiles between the two groups. However, at the peak time, a difference of 1.5-fold was observed in favor of the G-CSF group, with 3.80 ± 1.78 for the treated group and 2.47 ± 0.4 for the control group (*p* = 0.138) (Figure 2A,B).

### 2.5. Strong Temporal Modification of Vascularization in the Distracted Callus Induced by G-CSF

**Figure 3:**
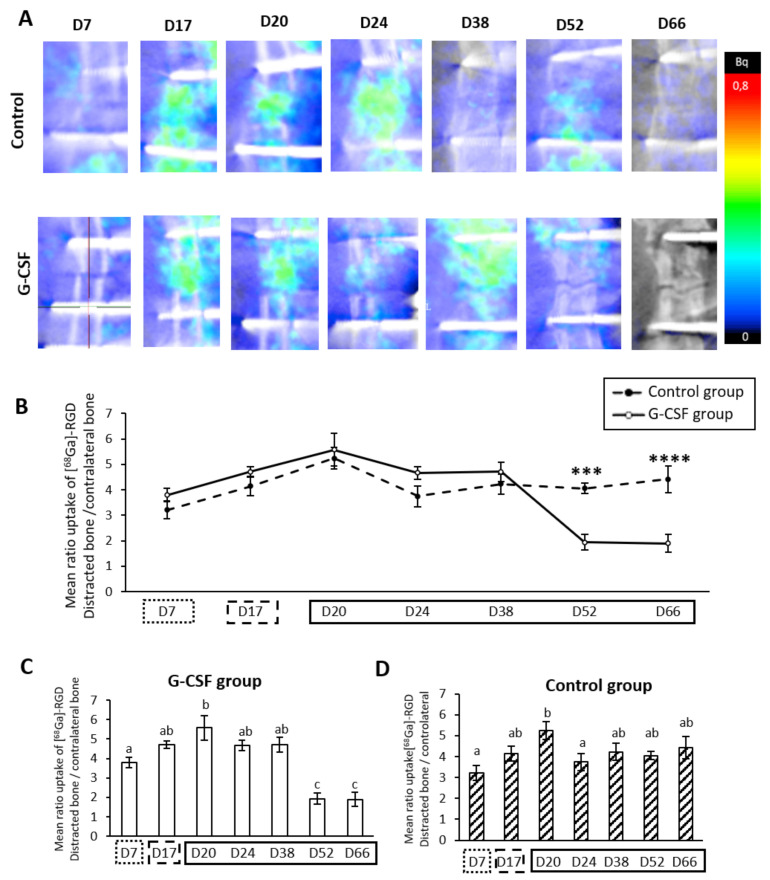
Course of vascularization metabolism during distraction osteogenesis (DO). (**A**) Representative positron emission tomography (PET) imaging 7, 17, 20, 24, 38, 52 and 66 days after surgery. (**B**) The temporal pattern of the mean uptake ratio of [^68^Ga]-RGD is presented and demonstrates vascular metabolism inside the new regenerate. The dotted line represents the control group and the filled line represents the G-CSF group. (**C**) In the G-CSF group, the mean ratio peaks at D20 compared to D7 (*p* = 0.007) and drops significantly at the end (*p* = 0.0001). (**D**) In the control group, a peak at D20 is also shown compared to D7 (*p* = 0.003) but only there is only a drop at D24 compared to D20 (*p* = 0.05). A PET study was performed on 6 control animals and 4 G-CSF animals. a, b, c: if two means share the same letter, no statistical differences were found. 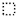 represent the latency period, the distraction phase is represented by 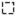, and 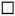 the consolidation phase. *** *p* < 0.01, **** *p* < 0.001.

We followed the vascular metabolism of the distracted callus using positron emission tomography (PET). The radiopharmaceutical gallium^68^ paired to the RGD sequence allowed the quantification of activated endothelial cells of vascularization via the integrin α_ν_β_3_. At the end of the latency period (D7), both groups showed a strong increase (3.38 ± 0.4 and 3.79 ± 0.3 for control and GCS-F groups, respectively) in the uptake ratio. No statistical difference was observed until D38 between the two groups. During the distraction phase, a marked increase in vascular activity was observed for the two groups. Four days after cessation of mechanical tensile forces (D20), a statistically different vascular peak was reached for both the control group (5.53 ± 0.6) and GCSF group (5.57 ± 0.6) (Figure 3C). For the control group, after this vascular peak, a significant drop to 3.65 ± 0.6 was apparent at D24, with a return in vascular activity values comparable to D7 that was maintained until D66 (Figure 3C), whereas, in the G-CSF group, no statistical difference was seen in the mean uptake ratio of [^68^Ga]-RGD at D24. In the latter group, a plateau was present up to D38 (4.71 ± 0.4). However, a strong significant drop of vascular activity was observed after D52 and D66 (1.94 ± 0.3 and 1.9 ± 0.3 for D52 and D66, respectively, *p* < 0.0001) (Figure 3C). At this time, the values were statistically different to those obtained for the control group (*p* < 0.01 and *p* < 0.001 for D52 and D66, respectively) (Figure 3B).

### 2.6. Histological Study

**Figure 4:**
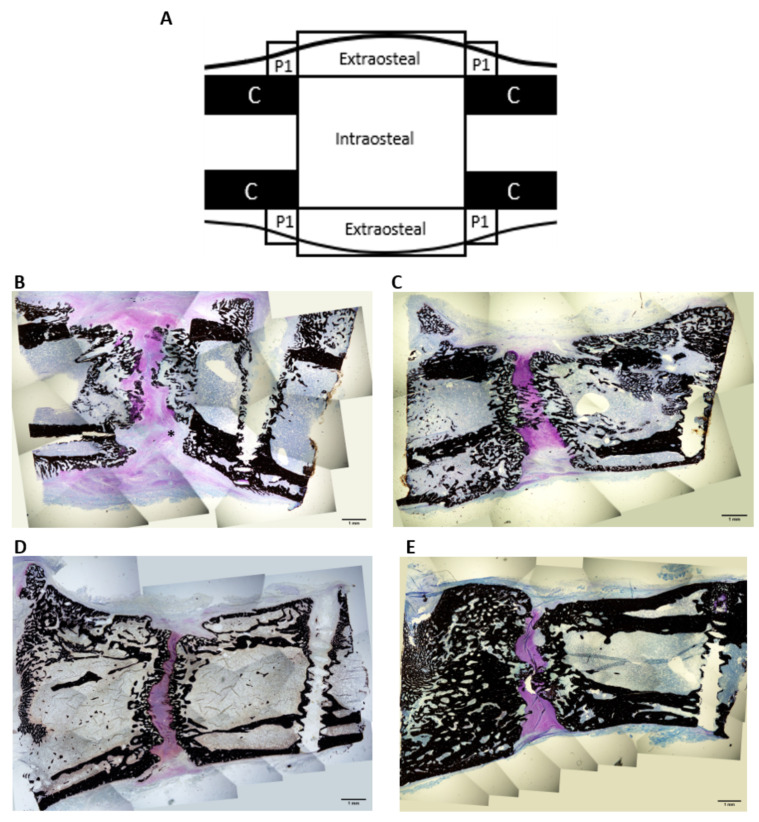
Histological images of the distracted callus at low magnification (×4) 31 days post-surgery (**B**,**C**) and 66 days post-surgery (**D**,**E**) in control group (**B**,**D**) and G-CSF group (**C**,**E**). (**A**) Region of interest (ROI) diagrams for histomorphometry analyses. (**B**) In the intraosteal region, fibrous tissue is still present. Note that the trabecular network visible is thinner and less connected than in the G-CSF group. In the central zone, the mineralized tissue is mostly composed of calcified cartilage rather than osseous tissue. (**C**) A bony bridge is apparent inside the intraosteal region. An intense cartilaginous zone is seen in between the two fronts of mineralization. The trabecular network is more developed and thicker. (**D**) The gap between the two osteotomized femur extremities is filled with cartilaginous tissue. No bridging is observed. In the periosteal zone, the double cortices are separated by a large medullary space. (**E**) The two bony ends of the femur are connected by a bony bridge in the center of the gap. In the periosteal zone, the bone marrow fills a small space, the cortices are thick and sometimes merged. Histological analysis was performed on 4 control animals and 4 G-CSF animals at each time. * = fibrous tissue.

Fourteen days after the distraction was stopped (D31), no bony bridging was seen in any ROIs (Regions of interest) in the control group (Figure 4B). The center of the intraosteal zone was mostly composed of calcified cartilage, cartilage tissue and some fibrous tissue. Closer to the native cortices, a trabecular network was apparent. In the G-CSF group, bony bridging was apparent in the center of the intraosteal area (Figure 4C). This zone was mostly composed of cartilage tissue, trabecular bone and calcified cartilage. As we approached the native cortices, the trabecular network was more dense and more connected compared to the control group. The latter was significantly thicker for the G-CSF group (0.081 ± 0.007, *p* < 0.05) compared to the control group (0.061 ± 0.007) (Table 1). Concerning the periosteal region, no difference between groups was observed for the area fraction or thickness of mineralized tissue. In each group, a second cortex formed along the native ones. Regarding the extraosteal region, even though no statistical difference was seen between groups, the mean values for the G-CSF group were higher, including 2-fold higher for the BA/TA (area fraction) and 1.3-fold higher for the thickness of the mineralized tissue compared to the control group (Table 1).

At 49 days of consolidation (D66), in the control group, although the mineralization fronts of the osteotomized bone were closer together compared to D31, still no bony bridging was seen (Figure 4D). However, except for the periosteal area, BA/TA mean values and trabecular thickness were significantly higher than those observed at 14 days of consolidation (D31). In the G-CSF group, BA/TA mean values and trabecular thickness were significantly higher at 49 days of consolidation (D66) compared to those observed at 14 days of consolidation in intraosteal, extraosteal and periosteal areas (Table 1). However, even though the mineralization fronts of the osteotomized bone got closer together, there was no noteworthy difference of the bony bridging compared to D31 (Figure 4E). Compared to the control group, intraosteal and periosteal BA/TA mean values as well as intraosteal trabecular thickness were significantly higher in the G-CSF group (Table 1).

**Figure 5:**
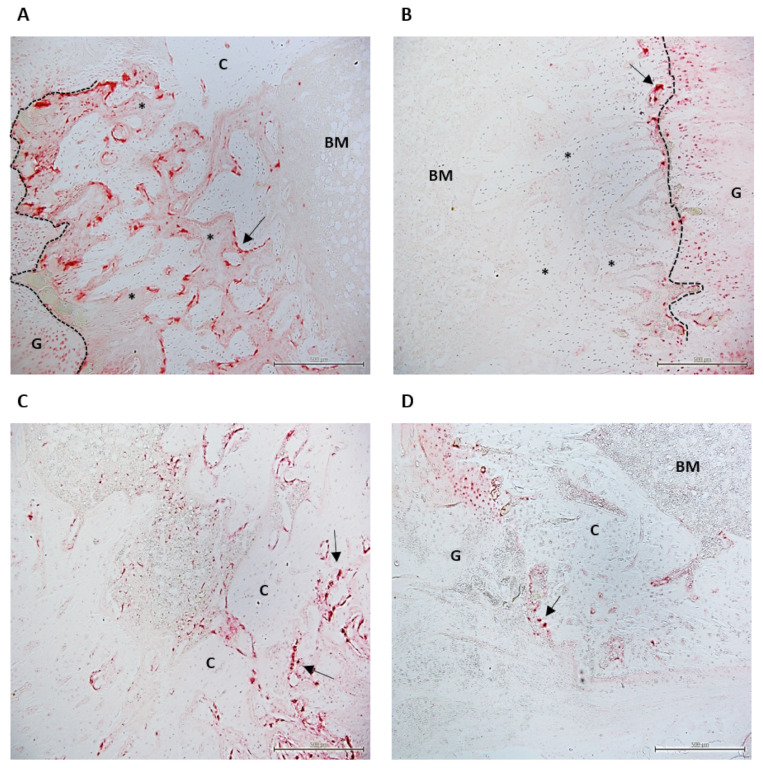
TRAP images of the distracted callus at low magnification (×10) 31 days (**A**,**B**) and 66 days (**C**,**D**) post-surgery in control (**A**–**C**) and G-CSF groups (**B**–**D**). At D31, in the control group, osteoclastic activity (arrow) is very high in the newly formed trabeculae (*) behind the mineralization front (dotted line) in the intraosteal area. In this area, in G-CSF animals, osteoclastic activity is restricted to the mineralization front (arrow). At D66, while osteoclastic activity is still significant in the extraosteal zone (arrow), this activity is minimal in this area in G-CSF animals (arrow). TRAP analysis was performed on 2 animals in each group at D31 and D66. C, cortical shaft; G, gap; BM, bone marrow.

At D31, the surface area (mm^2^) of the TRAP-positive osteoclasts, representing osteoclast activity, was different between the groups only for the intraosteal ROI where osteoclast activity was significantly higher for the control group compared to the G-CSF group (*p* < 0.05) (Table 2). Whereas osteoclastic activity was restricted to the mineralization front in G-CSF animals, it was largely developed on the newly formed trabeculae located between the bone marrow and the mineralization front in control animals (Figure 5). At D66, a significant difference (*p* < 0.05) was only observed in the extraosteal zone, with a lower osteoclast activity in the G-CSF group compared to the control group (Table 2, Figure 5).

## 3. Discussion

The purpose of this study was to determine the effects of systemic administration of low doses of G-CSF on bone regeneration in a rat model of DO and whether these effects were associated with a mobilization of osteo and vascular progenitor cells to highlight one possible mechanism of G-CSF action in a bone regenerative process. To our knowledge, this study is the first to show that systemic administration of low doses of G-CSF stimulates the bone formation and mineralization of the distracted callus, especially in the early phase of the consolidation. 

Endochondral ossification is one of the two bone-forming processes in DO and consists in the formation of a cartilaginous callus. As distraction begins, the cartilaginous callus first becomes hypertrophic and new bone formation occurs [42,43]. In this work, we conducted histological studies to monitor tissue formation inside the distracted callus. We found bone formation by endochondral ossification in the early phase of consolidation in control animals. Indeed, after 2 weeks of consolidation (D31), the intraosteal zone was composed of calcified and non-calcified cartilage tissue as well as newly formed bone. In addition, numerous points of endochondral ossification were found along the ossification front in the osteotomized region. These results are in accordance with the studies of Sato et al. [44] and Aronson et al. [45], in which endochondral ossification was described after 2 weeks of consolidation (D31). G-CSF did not change the predominant pattern of bone formation in the osteotomized region since cartilaginous tissue and endochondral ossification points were still present in the intraosteal zone of the callus. However, in treated animals, G-CSF induced the formation of a denser and more connected trabecular network with significantly thicker trabeculae compared to the control group. This network was sufficiently developed to form a bone bridge in the center of this region, indicating an acceleration of bone formation in G-CSF group. Moreover, although not significant, BA/TA was increased in the G-CSF group, particularly in the extraosteal area, with a 2-fold increase in treated animals compared to the control group. The surface occupied by the mineralized cartilage was more important in the control group than in the G-CSF group, and this probably explains the absence of a significant difference between the two groups since the surface occupied by the mineralized cartilage is taken into account in the assessment of BA/TA.

The osteoclastic response was also a strong evidence which supports the fact that G-CSF accelerated bone regeneration, especially in the distraction gap of the callus in the early phase of consolidation. As it has been established that the further along in the consolidation process, the more osteoclasia is reduced [21], the lower osteoclastic activity observed in the G-CSF group suggests that the consolidation process was advanced compared to the control group. Another histological observation supported this idea. In the intraosteal zone, we found osteoclastic activity of the two groups located at the front of mineralization. This is related to the fact that during endochondral ossification, the osteoclasts are needed for the degradation of the mineralized cartilage secreted by hypertrophic chondrocytes [46]. However, while osteoclastic activity was very high in the newly formed trabeculae behind the mineralization front in the control group, it was largely absent in the G-CSF group. Since mineralized tissue resorption precedes bone apposition during the regenerative process [46], we assume that the remodeling process of these bone trabeculae was more advanced and that trabeculae were therefore more mature, as evidenced by their greater thickness. Although other studies have already investigated the impact of G-CSF on bone regeneration as early as 2 weeks after bone injury [40,47,48,49], our study is the first to highlight the beneficial effect of low doses of G-CSF in DO. This beneficial effect of G-CSF on bone regeneration was still visible after 49 days of consolidation. In the control animals, although bone formation within the callus improved from D31 to D66, it was not sufficient to lead to bone bridging. These results are consistent with the literature. After 8 weeks of consolidation, studies with a distraction protocol similar to our study have described a not completely mineralized callus containing cartilaginous tissue [50] and no bridging between the osteotomized cortices [51]. In the animals treated with G-CSF, a larger amount of bone was present in the distracted callus compared to the control group. Indeed, in the intraosteal area, BA/TA was higher than in the control group and was associated with a denser trabecular network thanks to thicker bone trabeculae. In the periosteal area where BA/TA was also higher, the process of the formation of a unique cortex was far more advanced in the G-CSF group than in the control group. The higher osteoclast activity in the control group elicited by a larger surface area of TRAP-positive octeoclasts was also in favor of a better consolidation in G-CSF-treated animals than in control animals at 49 days of consolidation. These results demonstrated that the acceleration of bone regeneration after 14 days of consolidation persists in the late phase of consolidation. Liu et al. [47], Hermann et al. [40] and Moukoko et al. [39] have already shown the positive effect of G-CSF in late consolidation phases in segmental bone defect and bone fracture models.

To have a more global view on the kinetics of bone regeneration throughout the DO process, we longitudinally analyzed the deposition of newly formed hydroxyapatite crystal via planar scintigraphy, reflecting mineralization activity by osteoblasts. We found that a significant peak value was reached 3 weeks after cessation of the distraction forces (D38) regardless of the group. Mineralization during osteogenic distraction has already been studied by other authors [52,53,54,55,56] who reported that callus mineralization occurred predominantly after cessation of the distraction forces. In particular, Leung’s study [54] suggests that the mineralization activity is maximal in the early phase of consolidation since alkaline phosphatase activity, which is involved in bone and cartilage mineralization [57], peaked around 2 weeks after the end of the distraction phase (D31). Our scintigraphy data are in accordance with the latter study.

While G-CSF did not impact the temporality of tissue mineralization, it seems to impact the amount of crystal formation from 1 week of consolidation (D24) until the end of consolidation. Indeed, at 3 weeks of consolidation (D38), although there was no significant difference between the groups, the mean uptake ratio of [^99m^Tc]-HDMP was 1.5-fold higher in the G-CSF group than in the control group. This trend in increasing mineralization activity supports that G-CSF improved bone consolidation. The second hypothesis of our work was that mobilization of both osteogenic and vascular progenitor cells from bone marrow niches was one of the underlying mechanisms of G-CSF’s effect on bone regeneration during DO. Mesenchymal stem cells (MSCs) are cells that can differentiate into fibroblasts, chondroblasts or osteoblasts, each participating at their own level in the regeneration process in the different stages of bone repair [7]. As it has been shown that low doses of G-CSF can mobilize MSCs [58,59], we studied the mobilization of these cells. In our study, we characterized the MSCs using classical markers such as CD44+, CD90+, CD73+ and CD45− in accordance with the International Society for Cellular Therapy [20]. In the control group, we found an early increase in the number of circulating MSCs at the end of the latency period followed by a decrease after 4 days of lengthening, and from which mobilization increased for up to 2 weeks of consolidation (D31). This result confirms an early mobilization of MSCs during the DO process that has been recently reported by Yang et al. [60], who found a similar kinetic pattern of circulating MSCs during DO. Interestingly, in our study, the tensile forces induced by bone lengthening seemed to have no effect on the number of circulating MSCs, suggesting that mobilization of these cells is more dependent on inflammatory events induced by traumatic osteotomy than on mechanical events. Our work showed, for the first time, that G-CSF administration altered MSC mobilization during DO. However, unexpectedly and contrary to our hypothesis, the mobilization of MSCs was reduced with G-CSF since the number of MSCs was significantly lower at the end of the latency period compared to the control group. This surprising result is not in accordance with the literature since Kassis et al. [58] and Ripa et al. [59] demonstrated the mobilizing effect of G-CSF on MSCs. However, in the latter studies, the mobilizing effect was, respectively, determined in healthy subjects and subjects with acute myocardial infarction. In addition, the contribution of bone marrow-derived MSCs to the bone regeneration during DO has been shown in different studies. Indeed, following transplantation of these cells, Kitoh et al. [61], Qi et al. [62] and Yang et al. [60,63] reported an improvement in callus regeneration, probably related to the increase in homing of MSCs into the callus [22]. In view of these results, we believe that the lower counts of MSCs in the G-CSF group at the end of the latency phase could be explained by a peak of mobilization which occurred earlier. The study of Ripa et al. [59] supports our hypothesis. The latter described a very early mobilization of some sub-types of MSCs between 4 and 7 days after G-CSF administration. If so, it would support the idea that the acceleration of bone repair that we observed in the early phase of consolidation in the G-CSF group by histological, histomorphometry and scintigraphy studies could be favored by a very early mobilization of MSCs from bone marrow induced by the G-CSF. Further investigations should incorporate detection of these cells in the first days after G-CSF administration to confirm this hypothesis. Among MSCs, we also characterized a population of CD271^+^ MSCs that are less differentiated and that have been shown to preferentially differentiate into chondrogenic cells [64]. Our results showed that these cells, whose mobilization increased regardless of the group, represent a small sub-population of MSCs. G-CSF did not induce any modification in the kinetic pattern of these CD271+ MSCs, suggesting that these less differentiated MSCs are indifferent to the action of G-CSF.

In addition to bone and mineral matrix deposition, the maturation of the callus during DO requires bone resorption by osteoclasts. The latter are essential to convert cartilage to bone and ultimately remodeling of the callus [21]. Thus, in addition to MSCs, we also focused our attention on HSPCs since these cells provide precursors to osteoclasts [65]. The mobilization of HSPCs has never been studied during distraction osteogenesis. Our results showed that DO led to an increasing mobilization of HSPCs (CD34+, CD 45low between the latency and consolidation periods. Their number was significantly higher after 2 weeks of consolidation (D31) compared to the latency period and to the beginning of the distraction phase. The recruitment of these cells is most likely responsible for an increase in the number of osteoclasts in the callus as evidenced by the high osteoclastic activity previously described in our histological study after 14 days of consolidation. G-CSF injections altered the pattern of HSPC mobilization during DO. Indeed, four days after the beginning of elongation, HSPC mobilization was more than twice that in the control group. The mobilizing effect of G-CSF on HSPCs is known in healthy human subjects. G-CSF is used clinically to reduce chemotherapy-induced neutropenia and to treat severe chronic neutropenic disease as well in healthy donors to induce mobilization of CD34+ progenitor cells for transplantation [30,31]. This effect was also reported in healthy rats at higher doses (50 µg/kg) where mobilization of CD34+ and CD45+ cells was significant from the first day after the last G-CSF injection, with maximum mobilization at day 11 [40]. Therefore, it appears that the administration of G-CSF associated with bone lesions and the resulting inflammatory processes advanced the peak of mobilization. In our study, we observed this peak 4 days after the last injection of G-CSF with a dose of 5 µg/kg. In segmental bone defects in rabbits, Ishida et al. [49] even reported a peak of CD34+ as early as the first day after local application of a hydrogel containing G-CSF (5 µg).The increased availability of HSPCs, at the onset of elongation in the G-CSF group, is probably responsible for an earlier increase in osteoclast precursors compared to the control group, which argues in favor of G-CSF-accelerated bone regeneration, as demonstrated by our histological study.

Although the HSPC pool includes osteoclast precursors, it also contains EPCs. In order to specifically characterize this population, we used an EPC-specific marker (CD309) [20]. While injection of G-CSF altered the HSPC mobilization pattern during DO, it had no effect on the mobilization pattern of EPCs. Regardless of the groups, we found a significant increase in the number of EPCs during the distraction phase of DO, as previously reported by Lee et al. [25], highlighting the role of tensile forces in the mobilization of EPCs that has been evidenced by those authors. Since the participation of bone marrow-mobilized EPCs in neovascularization of injured tissue was clearly demonstrated [20], we also investigated the formation of new blood vessels in the distracted callus during DO. In both groups, we observed a 3- to 4-fold higher uptake of [^68^Ga]-RGD in the distracted callus than in the non-operated limb, indicating an active formation of new blood vessels in the callus at the end of the latency phase. Neovascularization of the callus was maintained at this level during the lengthening phase and during the early phase of consolidation. This temporal pattern of neovascularization of the callus was consistent with earlier results in the literature [8,25]. Interestingly, the increase in the number of EPCs was temporally related to the formation of new blood vessels in the distracted callus. These observations suggest a plausible contribution of bone marrow-mobilized EPCs to the increased neovascularization during distraction and early consolidation phases of DO, which had also been suggested in a previous work [25]. Contrary to our hypothesis, G-CSF neither promoted the mobilization of EPCs nor the neovascularization of callus during DO. The mobilizing capacity of G-CSF in EPCs has been demonstrated in a model of hindlimb ischemia [66] and a model of large bone defects [49]. Discrepancies in the G-CSF doses and in the method of its administration could account for the differences between our results and those of these two previous studies since the latter, respectively, used a five-fold higher dose [66] and a local administration of G-CSF at the injured sites [49]. Regarding neovascularization of the callus, surprisingly, during the late phase of consolidation, a significantly higher mean uptake ratio of [^68^Ga]-RGD was observed in the control group compared to the G-CSF group, while we found no difference in the mean uptake ratio of [^68^Ga]-RGD between the G-CSF and control groups until the early consolidation phase. In the only study that investigated vascularization in the late phase of consolidation in a dog DO model, blood flow was still two to three times higher than in the non-operated limb for up to 14 weeks of consolidation and persisted throughout the consolidation period, similar to the plateau we observed during this period in the control group. We hypothesize that the 2-fold lower neovascularization in the G-CSF group after 6 and 7 weeks of consolidation compared to the control group could be related to a very early peak in neovascularization induced by G-CSF between osteotomy and the end of the latency phase since Minamino et al. [66] showed a significant increase in the number of blood vessels in ischemic limbs as early as 3 days after the first injection of G-CSF. Such a very early peak of neovascularization, that our study design failed to evidence, could support, in part, the more advanced consolidation process of the callus we observed in the G-CSF group at D31 and D66 and account for a lower neovascularization in the late phase of consolidation.

## 4. Materials and Methods

### 4.1. Animal Model and Mobilizing Agents

All experiments were performed in accordance with the University of Aix-Marseille institutional animal care and use committee and the French Research Ministry. Animals were housed in individual cages, in a 12 h light/dark cycle and temperature-controlled room. Rats were closely monitored during the entire experiment and were fed a standard laboratory diet ad libitum. Twenty male Sprague Dawley rats (500 g ± 20), 12 weeks old at the day of surgery, were randomly divided into two groups. According to an established protocol, a procedure of distraction osteogenesis was applied on the right femur [67]. Briefly, the control and the treated group were assigned to a surgery of callotasis which consisted in setting the external fixator into the femoral bone and inducing a fracture with an oscillating saw. During the latency period (7 days), the animals of the treated group (*n* = 10) received 5 daily subcutaneous injections of 5 µg/kg per day of G-CSF (FILGRASTIM^®^, AMGEN^®^, Thousand Oaks, CA, USA) in the aftermath of surgery. Rats from the control group (*n* = 10) were injected subcutaneously for 5 days with a saline solution instead. Then, a 10-day period of distraction began, where the fixator was activated at a rate of 0.25 mm/12 h for a total elongation of 5 mm. At the end of the distraction phase, the fixator was locked, and the animals went through the consolidation phase for 49 days (D66). Figure S1 summarizes the DO protocol and indicates the methods used for the study of progenitors as well as for the monitoring and characteristics of the bone distracted callus. According to the recommendations of the ethics committee, different methods were performed on the same animal.

### 4.2. Phenotype Characterization by Flow Cytometry Analysis

Blood samples were collected in EDTA tubes (10.8 µL of 50 mg/mL EDTA) from the treated group (*n* = 6) and control group (*n* = 4) since two animals died in the control group during anesthesia. Samples were collected at 6 different time points: before surgery (D-2) and 7, 11, 17, 24, 31 days after the surgery (D7, D11, D17, D24, D31) for stem and progenitor cell counts. A halogenated isoflurane anesthesia with 3% induction and a 1.5% maintenance phase was applied to collect 300 µL of blood from the tail vein. After collection, the peripheral blood was divided into 3 aliquots of 100 µL for the characterization of three populations of stem cells: HSPCs, EPCs and MSCs. A panel of monoclonal and polyclonal antibodies were used for characterization of the immunophenotype of HSPCs, EPCs and MSCs: CD90 FITC (Biolegend^®^,San diego, CA, USA), CD271 PE (Thermo Fisher Scientific^®^, Waltham, MA, USA), CD73 A594 (Cliniscience^®^, Nanterre, France), CD45 APC/Cy7 (Ozyme^®^, Saint-Cyr-l’École, France), CD309 A488 (Cliniscience^®^, Nanterre, France), CD34 ECD (Beckman Coulter^®^, Villepinte, France), CD44 A647 (Ozyme^®^, Saint-Cyr-l’École, France). We used Rabbit-IgG Isotype control A488 (Cliniscience^®^, Nanterre, France) as an isotype control for CD309. Antibodies were added as a cocktail and aliquots were incubated on ice for 30 min. Then, to lyse red blood cells, each aliquot of 100 µL was treated with red blood cell lysis buffer, at a ratio of 1:10 on ice for 15 min. All sample preparations for this assay were done simultaneously. Flow cytometry markers are summarized in Supplementary Table S1 for HSPCs [68,69,70,71], MSCs [22,58,72,73,74,75,76,77] and EPCs [23,78,79,80,81]. The gating strategy is also presented in Supplementary Figure S2. Subsets of circulating blood cells were measured with a Cytoflex LX flow cytometer (Beckman Coulter^®^, Villepinte, France). Flow cytometry analyses were performed with at least 100,000 events per sample recorded.

### 4.3. Longitudinal In Vivo Positron Emission Tomography Measurements

The vascular metabolism of the distracted callus was longitudinally evaluated at 7 different time points after the surgery of callotasis (D7, D17, D20, D24-, D38, D52 and D66) via positron emission tomography (PET). The radiopharmaceutical gallium^68^ paired to the RGD sequence allowed the quantification of activated endothelial cells via the integrin α_ν_β_3_. A total of 6 animals in the control group and 4 in the G-CSF group were used for PET analysis. The PET data could not be collected for two G-CSF animals due to technical issues with the microscanner. The animals were anesthetized under 1.5% isoflurane, allowing the intraperitoneal injection of 10 ± 0.5 MBq of [^68^Ga]-RGD. One hour after the administration, animals were anesthetized a second time with the same dose for image acquisition with Mediso-NanoPET-CT (Mediso^®^, Budapest, Hungarian). Rats were positioned in the CT and the field of view was centered on the distracted callus. The voltage of the X-ray source was set to 50 kV and for an amperage of 980 µA. The frame time was 300 ms, giving a total scan time of 4 min. After scan acquisition, dynamic PET emission data were collected. The CT scan and PET images were then transferred to Vivoquant^TM^ software (Invicro^®^, Tokyo, Japan) to quantify radioactivity in the region of interest (ROI). No co-registration was necessary because the position of the animal was not changed as the imaging process was acquired sequentially. The selection of the ROI was made so that the distracted gap was fully included. The selected shape was cubic (10 × 10 × 10 = 1000 mm^3^) and located between the two native cortices. The same ROI was used for the contralateral femur at the same location. This second ROI allowed us to normalize our values of vascular activity and to overcome intermanipulation bias. The tissue uptake values given by the PET analysis are presented as the mean ratio [^68^Ga]-RGD uptake.

### 4.4. Longitudinal In Vivo Scintigraphy Analyses

Mediso-NanoSPET-CT (Mediso^®^, Budapest, Hungarian) was used to provide planar SPECT images to longitudinally monitor the continuous deposition of hydroxyapatite (CA_10_(PO_4_)_6_(OH)_2_) from the beginning of the regenerative process to the end of the study. In fact, the accumulation by chemisorption of the radiotracer [^99m^Tc]-HDMP onto newly formed hydroxyapatite crystals allowed us to longitudinally monitor the osteoblast activity inside the distracted callus (Figure 3A). Each planar SPECT imaging session occurred after a 24 h delay after PET imaging to prevent background signals and the same panel of animals was used during the scintigraphy assay (4 in the treated group and 6 in the control group). Rats were anesthetized under 1.5% isoflurane, allowing the administration of 30 ± 1.5 MBq of [^99m^Tc]-HDMP into the tail vein via a catheter. Four hours after the systemic injection, rats were anesthetized a second time with an initial dose of 5% isoflurane that was diminished to 1.5% to maintain the anesthesia during the 10 min imaging process. The ROI selected for quantitative analysis of the planar SPECT images was also examined with Vivoquant^TM^ software (Invicro^®^, Tokyo, Japan). The selected ROI was chosen so that all of the femur was selected (starting from the femoral head to the femoral condyle) and a symmetrical ROI was selected on the contralateral femur, allowing us to calculate the mean uptake ratio of [^99m^Tc]-HDMP. As for PET imaging, the tissues uptake values were given as the mean ratio of the distracted bone to the contralateral bone.

### 4.5. Histological Analysis

Four of the distracted calluses were harvested for histological analysis at D31 and D66 (*n* = 2 for each group).

#### 4.5.1. Histomorphometric Analysis

The external device and the pins were removed. Then, the distracted callus was resized using a dental saw under constant hydration to avoid tissue damage so that only the callus with the adjacent cortical bone was kept. The samples were fixed in 70% ethanol for one week. Following fixation, tissue samples were washed with deionized water and were dehydrated through an ethanol gradient of 80%, 95%, 100% ethanol, each step lasting 48 h. Samples were cleared in methylcyclohexan (VWR international^®^, Radnor, PA, USA) for 48 h before infiltration and embedding in methyl methacrylate (MMA) resin (VWR international, Radnor, PA, USA). Samples then underwent polymerization in a 28 °C water bath for 3 days. After trimming of the blocks, 5 µm thick longitudinal sections were obtained using a microtome (Leica© RM 2265, Wetzlar, Germany) equipped with a D-profile tungsten carbide knife. The sections were transferred to Superfrost Plus slides. Before staining, MMA was removed from the sections by immersion in three changes of 2-methoxyethylacetate (Merk Corporation^®^, Burlington, MA, USA) for 20 min each, one change of ethanol 70 for 5 min, one change of ethanol 40 for 5 min and then rehydrated in two deionized water baths. The sections were stained with Von Kossa and counterstained with toluidine blue. They were dehydrated and mounted using a resinous mounting medium (Entellan^®^, Merk Corporation, Burlington, MA, USA). Quantitative 2D metrics were obtained on several ROIs (Figure 5A): (1) intraosteal and extraosteal ROIs, and (2) the periosteal area which represents the mean of the four P1 regions. Only the mineralized tissue was analyzed within the ROI and the metrics were mean bone area/total area (BA/TA) and mean trabecular thickness (Tb.Th). All measurements were evaluated by means of NIH image software [82] and the plugin BoneJ [83].

#### 4.5.2. TRAP Staining

Tartrate-resistant acid phosphatase (TRAP) is an enzyme that is found mostly in osteoclasts and currently used for the assessment of bone resorbing activity. Rehydrated sections were incubated in substrate solution at 37 °C overnight. The latter consisted of 1 mL naphtol AS-TR phosphate substrate mix (15 mg naphtol AS-TR phosphate dissolved in 1 mL N,N dimethylformamide) in 100mL acetate buffer (pH 5.0) enriched with sodium tartrate. The slides were then transferred without rinsing in revelation solution and incubated 10 min at 37 °C. This consisted of 1 mL sodium nitrite (4% in distilled water), 1 mL pararosaniline dye and 100 mL acetate buffer (pH 5.0) enriched with sodium tartrate. The sections were rinsed in running water for 5 min. They were dehydrated and mounted with a resinous mounting medium (Entellan^®^, Merk Corporation, Burlington, MA, USA). Control sections were incubated in a solution that did not contain the substrate. No staining developed in these control sections. All stained sections were examined under a light microscope (BX-40, Olympus^®^, Tokyo, Japan) equipped with a CCD camera (DP21, Olympus^®^, Tokyo, Japan). Surface area (mm^2^) of the TRAP-positive osteoclasts were also measured by means of NIH image software [82] with the help of the Trainable Weka Segmentation plugin [84].

### 4.6. Statistical Analysis

Collected data were expressed as mean values ± standard error of the mean (SEM). All statistical analysis was done using XLSTAT software (Addinsoft^®^, Paris, France). Before each statistical test, all the measured data were tested for normal distribution with a Shapiro test (*p* > 0.05). Multiple comparisons among group and time were tested with a repeated measures ANOVA using restricted maximum likelihood (mixed models). Those were followed by post hoc testing with the Fisher’s least significant difference procedure. A *p*-value < 0.05 was considered for statistical significance. For histological and TRAP analyses, a non-parametric Mann–Whitney *U* test was used since data did not follow a normal distribution. 

## 5. Conclusions

Our study suffers from a few limitations. First, it was carried out on a small sample size for control and G-CSF groups. Second, its design did not allow us to determine the homing of progenitor cells in the distracted callus. Last, it did not provide data to characterize a beneficial effect of G-CSF on the mechanical behavior of the distracted callus. Further studies should take into account these limitations to determine whether G-CSF could allow an earlier removal of the distractor and therefore a shortening of the surgical treatment, and to elucidate the mechanisms by which G-CSF enhances bone regeneration during distraction osteogenesis. Despite its limitations, this study showed that a low dose of G-CSF, as already used in clinical practice, accelerated bone regeneration in the early phase of consolidation during DO. Its potential use in therapeutics could thus reduce the many complications inherent to this surgical technique. Considering our results on the effects of G-CSF on the mobilization of progenitor cells, we believe that the improvement of callus regeneration with G-SCF is the result, in part, of both a very early increased mobilization of progenitor cells from the bone marrow and of a favored homing into the distracted callus.

## Figures and Tables

**Table 1:** Histological evaluation: area fraction (BA/TA) and 2D trabecular thickness. Values are given as mean ± SEM. Compared to control group, ^a^ *p* <0.05; compared to D31, ^b^ *p* <0.05.

ROIs		BA/TA (%) Mean ± SEM		Trabecular Thickness (mm) Mean ± SEM	
		Control Group (*n* = 2)	G-CSF Group (*n* = 2)	Control Group (*n* = 2)	G-CSF Group (*n* = 2)
D31	Intraosteal	0.260 ± 0.012	0.280 ± 0.027	0.061 ± 0.003	0.081 ± 0.003 ^a^
	Extraosteal	0.095 ± 0.074	0.189 ± 0.018	0.049 ± 0.010	0.063 ± 0.005
	Periosteal	0.272 ± 0.03	0.333 ± 0.04	0.074 ± 0.006	0.073 ± 0.005
D66	Intraosteal	0.366 ± 0.027 ^b^	0.464 ± 0.031 ^a,^^b^	0.119 ± 0.002 ^b^	0.148 ± 0.10 ^a,^^b^
	Extraosteal	0.304 ± 0.010 ^b^	0.344 ± 0.032 ^b^	0.127 ± 0.014 ^b^	0.123 ± 0.004 ^b^
	Periosteal	0.297 ± 0.018	0.506 ± 0.047 ^a,^^b^	0.126 ± 0.008 ^b^	0.145 ± 0.017 ^b^

**Table 2:** Tartrate-resistant acid phosphatase (TRAP) analysis. Values are given as mean ± SEM. Compared to control group, ^a^ *p* < 0.05.

ROIs		Area mm² Mean ± SEM	
		Control Group (*n* = 2)	G-CSF Group (*n* = 2)
D31	Intraosteal	0.593 ± 0.103	0.280 ± 0.047 ^a^
	Extraosteal	0.089 ± 0.045	0.099 ± 0.028
	Periosteal	0.042 ± 0.004	0.044 ± 0.016
D66	Intraosteal	0.04 ± 0.009	0.068 ± 0.017
	Extraosteal	0.059 ± 0.012	0.015 ± 0.006 ^a^
	Periosteal	0.002 ± 0.0005	0.017 ± 0.061

## Data Availability

The data presented in this study are available on request from the corresponding author.

## Supplementary Materials

Supplementary materials are available online at https://www.mdpi.com/article/10.3390/ijms22073505/s1.
