# Supplementary material for: Systemic Administration of G-CSF Accelerates Bone Regeneration and Modulates Mobilization of Progenitor Cells in a Rat Model of Distraction Osteogenesis
Source: Int J Mol Sci. 2021 Mar 28;22(7):3505. doi: 10.3390/ijms22073505 (PMC8037338; doi:10.3390/ijms22073505)
Supplement: Supplementary file 1 [file ijms-22-03505-s001.pdf]

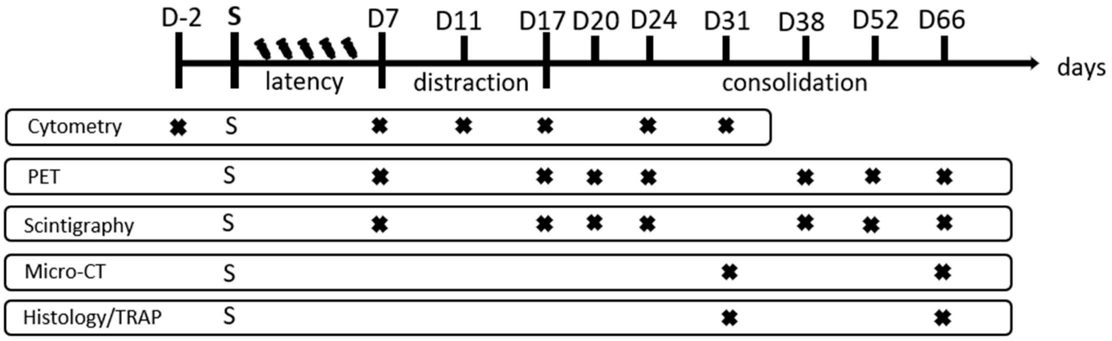

Figure S1: Representation of the experimental design. S = Surgery. Systemic injections of G-CSF and placebo are represented by [syringe symbol]. The crosses indicate the dates when the different analyses were performed.

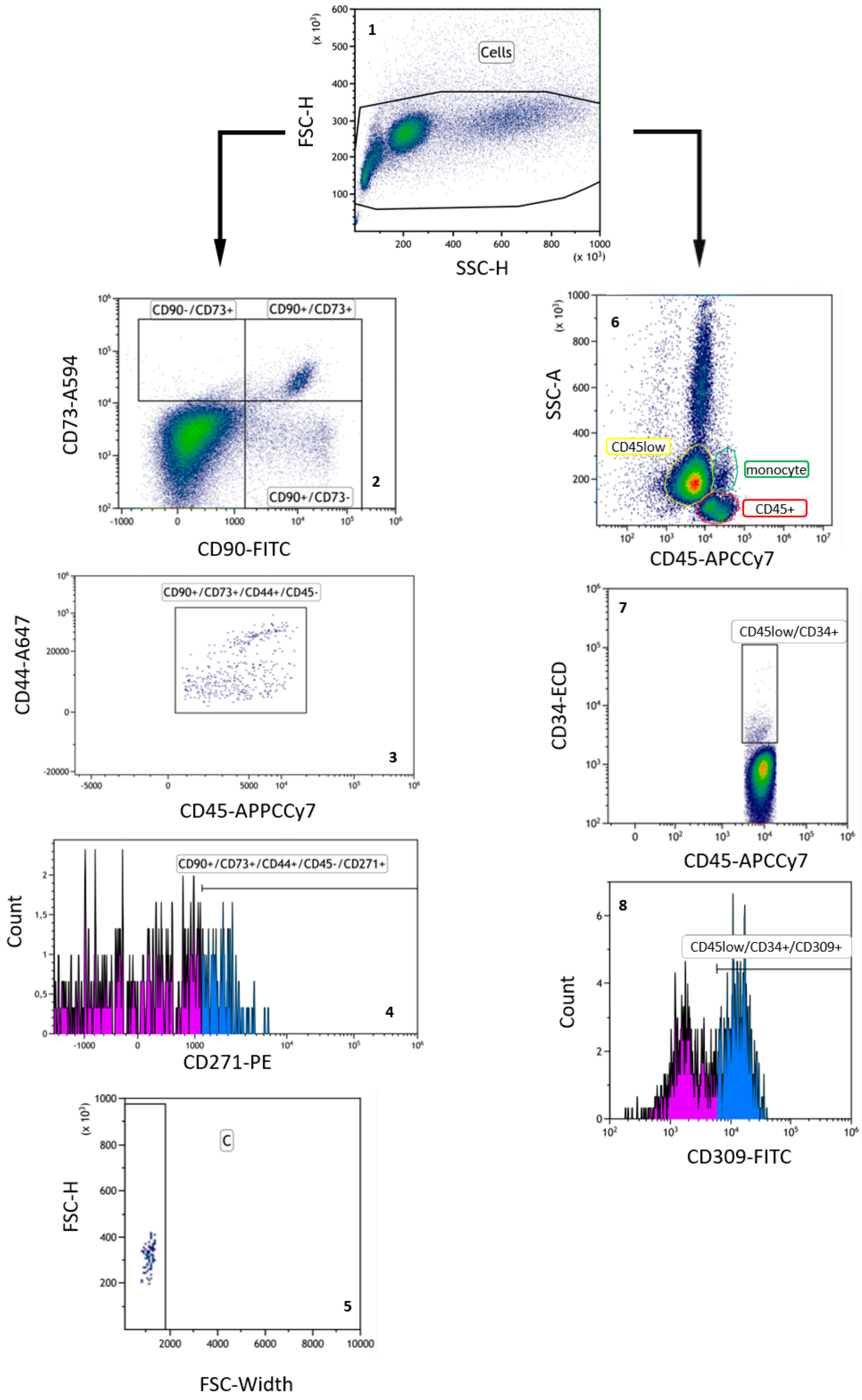

Figure S2. Representative flow cytometry gating strategy for each cell types depending on their immunophenotypic profile. Plot 2 to 5 represent the MSC gating, with plot 4 for MSC CD271+. Then, plot 7 represent the HSPC population and the plot 8 the EPCs cell type.

Table S1. Flow cytometry markers.

Cell type	Surface Markers	Comment	Dosage
HSPC	CD34+	Hematopoietic maker	10 μL no dilution
	CD45low	Leukocyte marker	2 μL no dilution
EPC	CD34+	Hematopoietic marker	10 μL no dilution
	CD45low	Leukocyte marker	2 μL no dilution
	CD309+	Endothelial marker	2 μL no dilution
MSC	CD44+	Express by most mammalian cells	3 μL diluted 20 times
	CD73+	Mesenchymal marker	4 μL no dilution
	CD90+	Mesenchymal marker	2 μL no dilution
	CD271+	Marker associated with stemness of MSC	4 μL no dilution
	CD45−	Leukocyte marker	2 μL no dilution
